# Effect of Resin Cement Viscosity and Thickness on the Shear Bond Strength of a Lithium Disilicate Glass-Ceramic

**DOI:** 10.1016/j.identj.2026.109474

**Published:** 2026-03-03

**Authors:** Renan Vaz Machry, Gabriel Kalil Rocha Pereira, Kiara Serafini Dapieve, Ana Carolina Cadore-Rodrigues, Amanda Maria de Oliveira Dal Piva, Luiz Felipe Valandro, João Paulo Mendes Tribst, Cornelis Johannes Kleverlaan

**Affiliations:** a Department of Restorative Dentistry, Federal University of Minas Gerais (UFMG), Belo Horizonte, Minas Gerais, Brazil; b Department of Restorative Dentistry (Prosthodontic Unit), Federal University of Santa Maria (UFSM), Santa Maria, Rio Grande do Sul State, Brazil; c Department of Dental Materials Science, Academic Centre for Dentistry Amsterdam (ACTA), Universiteit van Amsterdam and Vrije Universiteit, Amsterdam, Noord-Holland, The Netherlands; d Department of Reconstructive Oral Care, Academic Centre for Dentistry Amsterdam (ACTA), Universiteit van Amsterdam and Vrije Universiteit, Amsterdam, The Netherlands

**Keywords:** Cementation, Ceramics, Dental bonding, Dental cements, Shear strength, Viscosity

## Abstract

**Objective:**

To assess how resin cement viscosity and thickness influence the static shear bond strength (s-SBS) of adhesively bonded lithium disilicate ceramic.

**Methods:**

Cylindrical lithium disilicate samples were prepared and randomly assigned to four experimental groups (*n* = 10), based on two factors: resin cement thickness (thin ≈50 µm or thick ≈150 µm) and viscosity (high or low). A tri-layer configuration (ceramic–resin cement–ceramic) was used, with standardized ceramic discs obtained from CAD/CAM blocks. The bonding ceramic surfaces were etched with hydrofluoric acid, silanized, and a defined bonding area (≈6 mm^2^) was delimited using adhesive tape. The resin cement thickness was controlled by applying either one (thin) or three (thick) overlapping layers of adhesive tape. Resin cement was applied, and assemblies were seated under a 100 g static load. After light-curing, specimens were subjected to static shear bond strength testing (s-SBS) using a universal testing machine (crosshead speed: 1 mm/min; load cell: 1 kN). Failure mode was classified under stereomicroscopy, and representative samples were analysed under SEM. Additional specimens were used to confirm cement layer thickness using a profilometer.

**Results:**

For the thick resin cement layer (≈150 µm), viscosity significantly influenced bond strength, with high-viscosity cement showing higher mean values (27.36 MPa) compared to the low-viscosity cement (19.65 MPa). In contrast, resin cement viscosity did not affect the bond strength in the thin layer condition (≈50 µm). Most specimens exhibited adhesive failure at the ceramic–cement interface.

**Significance:**

Resin cement selection based on viscosity is particularly relevant when a thick cement layer is anticipated, whereas for ideal, thin cement films, viscosity is a less consequential factor for interfacial bond strength.

## Introduction

Factors such as resin cement thickness^1^, ^2^, ^3^, ^4^, ^5^ and viscosity^6^, ^7^, ^8^ can affect the adhesive and mechanical performance of resin-bonded lithium disilicate glass-ceramic restorations. These effects may result from differences in elastic modulus between ceramic – resin cement – substrate,^3^ the manner the luting agent fills and penetrates the defects on the etched ceramic surface, and the risk of occurrence of bubbles,^7^^,^^9^ and the proper seating of the restorations.^8^

There is a growing body of literature recognizing the important role of resin cement layer thickness in the mechanical behaviour of bonded restorations in lithium disilicate.^2^^,^^5^^,^^10^ According to the International Organization for Standardization (ISO), the layer thickness of luting materials should not exceed 50 µm.^11^ Despite that, discrepancies of up to 120 μm are frequently reported as clinically acceptable,^12^ and values exceeding both thresholds (50 or 120 μm) are commonly observed in studies.^10^

Indeed, lithium disilicate crowns resist higher loads when bonded with a 50 µm cement layer compared to a 500 µm layer.^2^^,^^3^ This finding is supported by studies indicating that thinner cement layers are significantly related to mechanical behaviour improvement.^1^^,^^4^ On the other hand, some evidence suggests that small differences in layer thickness may not substantially influence the stress distribution (60-120 µm,^5^ 100-300 µm),^13^ bond strength (60-120 µm),^5^ fracture resistance – static (50-100 µm,^2^ 50-100 µm),^4^ or fatigue performance (50-300 µm,^9^ 50-200 µm),^14^ of glass-ceramic assemblies.

In light of these contradictory findings regarding cement thickness, another significant aspect to consider is the viscosity of the resin cement, which must allow complete seating while maintaining a minimal and controlled cement layer. This allows complete seating of the restoration without excessive pressure, thereby preventing stress concentration in the ceramic structure.^15^^,^^8^^,^^16^ In addition, the use of resin cements with higher viscosity suggests a better filling of superficial defects created by the acid used to etch the ceramic adhesive surface, which can promote better results concerning bond strength.^17^ Despite that, few studies have investigated how cement thickness, influenced by the viscosity of different luting agents, affects the adhesive performance of lithium disilicate ceramics.

Thus, the question arises: can the resin cement-viscosity and thickness affect the bond strength to lithium disilicate glass ceramic? Therefore, this study aimed to explore the influence of different resin cement viscosities (high and low) and thicknesses (thin and thick) on their shear bond strength (s-SBS) to lithium disilicate. The conceptual hypotheses were: (1) resin cement viscosity will affect s-SBS results; (2) resin cement thickness will not affect s-SBS.

## Materials and methods

The characteristics of the materials used in this study are described in Table 1.

**Table 1:** Description of materials, commercial name, manufacturer, composition, and batch number.

Material	Commercial name/manufacturer	Composition*	Batch number
5% Hydrofluoric acid	IPS Ceramic Etching Gel/ Ivoclar, Schaan, Liechtenstein	<5% hydrofluoric acid	Y48112
35% Phosphoric acid	K-etchant syringe gel/ Kuraray Noritake Dental Inc., Okayama, Japan	Aqueous solution of phosphoric acid and colloidal silica	BV0048
Dual-curing resin cement	Variolink N High-Viscosity (Catalyst)/Ivoclar AG	Barium glass filler and mixed oxide, dimethacrylates, ytterbiumtrifluoride, initiators and stabilizers and pigments	YZ1262
	Variolink N Low-Viscosity (Catalyst)/Ivoclar AG		YZ1263
	Variolink N Base/Ivoclar AG		YZ1282
Lithium disilicate glass-ceramic	IPS e.max CAD, HT A2, C14/ Ivoclar AG	SiO_2_, Li_2_O, K_2_O, P_2_O_5_, ZrO_2_, ZnO, other and colouring oxides	Y52153
Silane-based coupling agent	Monobond N – Ivoclar AG	Alcohol solution of silane methacrylate, phosphoric acid methacrylate, and sulphide methacrylate	Y45831

⁎ The chemical composition is described according to the manufacturers’ information.

### Study design

**Fig. 1:**
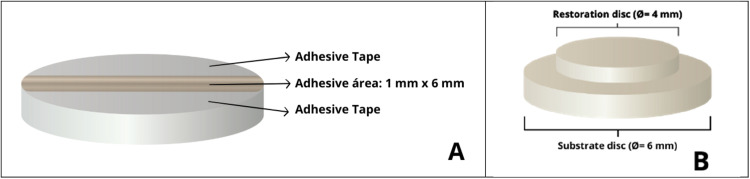
A, Representative schematic of the adhesive area, measuring 1 mm in width (delimited by adhesive tapes) by 6 mm in length (substrate disc diameter). B, Schematic of the sample assembly, showing the restoration disc (Ø = 4 mm) adhesively cemented onto the substrate disc (Ø = 6 mm), with the resin cement layer positioned between the two ceramic discs.

The experimental design was defined by four groups, considering two study factors: the ‘resin cement thickness’ in two levels: thin or thick; and the ‘resin cement viscosity’, also in two levels: high- or low-viscosity (*n* = 10, Table 2). For all groups, a tri-layer setup (ceramic – resin cement – ceramic) was used as described in.^7^ The use of lithium disilicate ceramic for both setup discs, referred to as substrate (Ø = 6 mm; *n* = 40) and restoration (Ø = 4 mm; *n* = 40), aims to ensure that adhesive failures were limited to the resin cement–ceramic interface (Figure 1). The ceramic discs preparation, surface treatments, and bonding procedures were performed by three trained operators (K.S.D, R.V.M, A.C.C.R) as follows:

**Table 2:** Experimental design (*n* = 10).

Groups’ codes	Resin cement	
	Viscosity	Layer thickness (approximately)
H-50	High	50 µm (thin)
H-150		150 µm (thick)
L-50	Low	50 µm
L-150		150 µm

### Ceramic discs preparation

**Fig. 2:**
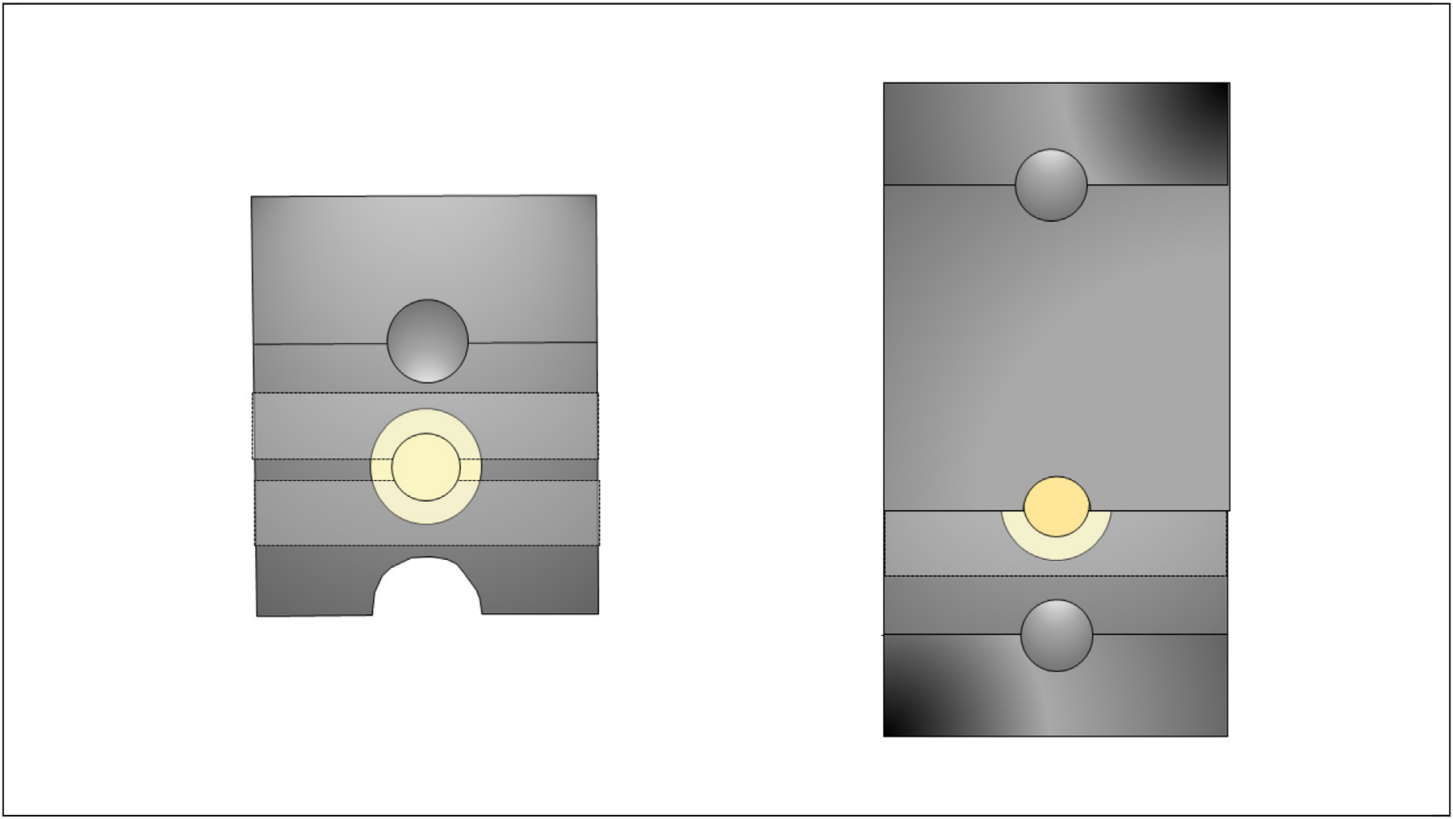
Sample setup (left) and testing device (right).^7^

Diamond drills (Ø = 6 mm and 4 mm; Diamant Boart, Brussels, Belgium) attached to a bench drill (SBE 1010 Plus, Metabo, Nürtingen, Germany) were used to shape ten lithium disilicate CAD/CAM blocks (IPS e.max CAD, Ivoclar AG, Schaan, Liechtenstein) into cylinders (five with Ø = 6 mm and five with Ø = 4 mm), under water cooling. Next, the cylinders were cut (≈1.6 mm in thickness) under water cooling (Isomet 1000, Buehler, Lake Bluff, United States) to create ceramic discs for substrates (Ø = 6 mm; *n* = 40) and restorations (Ø = 4 mm; *n* = 40). The discs were then polished using #400-, #600-, and #1200-grit SiC papers (CarbiMet SiC Abrasive Paper, Buehler) in a polishing machine (Ecomet, Buehler) to standardize the surfaces and achieve a thickness of 1.5 mm for both the substrate and restoration. Then, the ceramic discs were crystallized in a specific furnace (Programat P100, Ivoclar AG) according to the manufacturer’s instructions (10 minutes at 840 °C, 7 minutes vacuum). Subsequently, the ceramic substrates were embedded in a specific metallic device using cyanoacrylate for the shear bond strength test (Figure 2).^7^

### Surface treatments and bonding procedure

The embedded substrate discs (Ø = 6 mm) and restoration discs (Ø = 4 mm) were cleaned in an ultrasonic bath (distilled water; 5 minutes) and carefully air-dried before the bonding procedure.

The bonding surfaces of both substrates and restorations were etched with 5% hydrofluoric acid (HF) (IPS Ceramic Etching Gel, Ivoclar AG) for 20 seconds, rinsed with air-water spray for 30 seconds, cleaned in an ultrasonic bath (distilled water; 5 minutes), and air-dried for 30 seconds. Then, the silane-based coupling agent (Monobond N, Ivoclar AG) was applied for 60 seconds, following the manufacturers’ instructions.

The bonding area was delimited to 1 mm in width using adhesive tapes (Scotch Magic Tape, 3M, Saint Paul, United States), precisely positioned in parallel on the ceramic substrate surface with the aid of a digital caliper (Absolute Digimatic, Mitutoyo, Kawasaki, Japan), forming a linear bonding interface defined by the 1 mm spacing between the tapes and the disc diameter (∼6 mm) (Figure 1). One layer of tape was positioned to create a thin layer (50 µm), while three overlapping tapes were used to create a thick layer (150 µm). With the tapes in place and with the bonding surface untouched, the resin cement (Variolink N, Ivoclar AG) was prepared by mixing a single base component with either a high- or low- viscosity catalyst (1:1 ratio) and applied on the ceramic restoration surface. Each restoration was positioned onto the substrate disc, and the assembly was seated under a static load of 100 grams. The resin cement excess was removed with a micro-applicator, and the assemblies were light-cured (Elipar FreeLight 2, 3M) for two exposures of 20 seconds. After 24 hours of cementation, the embedded sets were aligned with their corresponding metallic analogue counterparts prior to mechanical testing (Figure 2).

To verify the thickness of the resin cement layer (50 µm or 150 µm), additional lithium disilicate substrates for each thickness and resin cement viscosity condition (*n* = 1) were bonded onto glass plates and detached. Thus, it was possible to measure the cement layer thickness for each condition in a profilometer (Mitutoyo SJ 400 Profilometer, Mitutoyo), as shown in Table 3. Measurements were taken in three regions of each sample to calculate the mean and standard deviation. The measurements confirm the planned thickness of the resin cement layers using one (thin) and three (thick) tapes, regardless of the resin cement viscosity.

**Table 3:** Mean and standard deviation (SD) of resin cement layer thicknesses (µm) and representative graphs obtained by profilometry analysis. It is possible to visualize the shape of the cement layer in a cross-sectional view.

### Static shear bond strength testing (s-SBS)

The s-SBS (*n* = 10) was performed in a universal testing machine (Instron 6022, Instron, Norwood, United States) at a crosshead speed of 1 mm/minute with a stainless-steel load applicator (Ø = 5 mm).^18^ The load to fail was recorded in Newton (N), and the s-SBS was calculated according to the formula: S = L/A, in which the bonding area was individually determined based on the distance between the adhesive tapes and the diameter of the ceramic substrate disc.

### Failure analysis

**Fig. 3:**
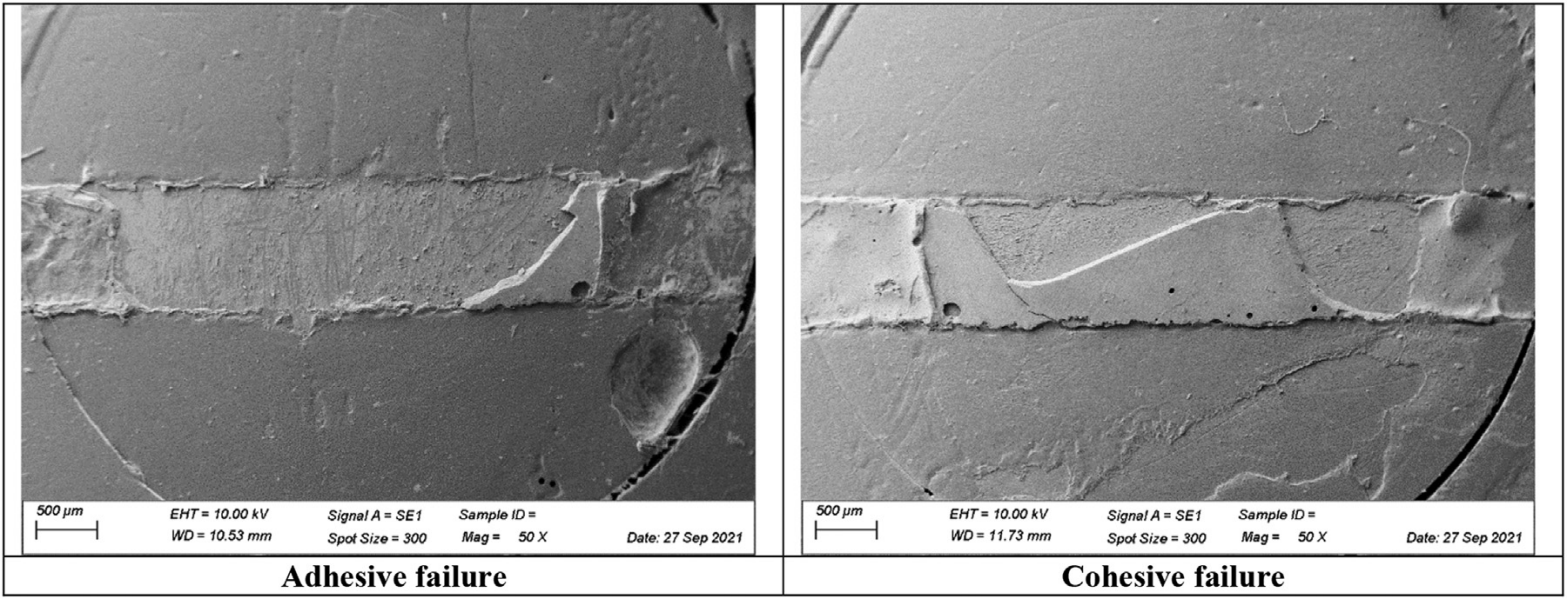
Representative microscopy images of the failure patterns (adhesive and cohesive). Cohesive failures were observed exclusively in the high-viscosity resin cement group with reduced film thickness, where entrapped air voids were detected. These voids may have acted as stress concentrators, contributing to the cohesive failure pattern and increased variability in shear bond strength values.

The fracture pattern was determined under a stereomicroscope (Olympus, Shinjuku, Japan), and classified into two types: (1) adhesive failure at the interfacial region between the resin cement and ceramic (adhesive areas up to 50%); (2) cohesive failure in the cement (cohesive areas from 50%). Representative specimens (adhesive and cohesive) were ultrasonically cleaned (distilled water, 5 minutes), air-dried, gold-sputtered (Edwards S150B, BOC Edwards, Burgess Hill, United Kingdom), and analysed under Scanning Electron Microscopy (SEM, Evo LS15, Carl Zeiss, Gottingen, Germany) at 50× magnification to observe the failure pattern (Figure 3). Failure mode classification was performed by a single calibrated examiner, following predefined criteria to ensure consistency and reduce intra-examiner variability.

### Data analysis

A statistical software program (IBM SPSS Software, IBM, Armonk, United States) was used to perform the data analysis, adopting a significance level of 0.05, disregarding the cohesive failures. Data were diagnosed as non-normal by the Shapiro-Wilk test (*P* = .03; *P* = .029; *P* = .152; *P* = .003) and as non-homoscedastic by the Levene’s test (*P* = .031). Although normality and homoscedasticity assumptions were violated, a factorial ANOVA (2 × 2) was used as the base model to evaluate the main effects and interaction between resin cement thickness and viscosity. Statistical inference was supported by bias-corrected and accelerated (BCa) bootstrapping procedures (1000 resamplings; 95% CI), which account for deviations from normality, heteroscedasticity, and unequal group sizes.^19^ Failure modes and topographic features were qualitatively analysed.

## Results

Two-way ANOVA showed a statistically significant effect for ‘resin cement viscosity’ factor (F = 10.743, *P* = .002), but only the thick layer was affected by viscosity, favouring the high (27.36 MPa) compared to the low-viscosity (19.65 MPa) (*P* = .043). The ‘resin cement thickness’ factor, in a representative range of 55.63 to 153.90 µm (F = 0.561, *P* = .459) and the ‘thickness*viscosity’ interaction (F = 0.434, *P* = .515) did not present a statistical influence. All groups exhibited 100% adhesive failure, except for the thin layer with high-viscosity resin cement (30% of cohesive failure) (Table 4), where entrapped air bubbles were evident (Figure 3) and likely contributed to the failure pattern and the greater variability in bond strength results, as reflected by the highest standard deviation in s-SBS values (Table 4).

**Table 4:** Mean and standard deviation (SD) of static shear bond strength (s-SBS; MPa)*, and failure types (adhesive failure; cohesive failure in the cement).

Thickness	High-viscosity			Low-viscosity		
	s-SBS	Failure types		s-SBS	Failure types	
		Adhesive	Cohesive		Adhesive	Cohesive
50 µm	31.51 (13.98) ^A^^a^	7 (70%)	3 (30%)	19.91 (3.61) ^Aa^	10 (100%)	-
150 µm	27.36 (8.87) ^Aa^	10 (100%)	-	19.65 (8.09) ^Ab^	10 (100%)	-

^Aa,Ab^Statistical differences are represented by uppercase letters in the column (compare thickness) and lowercase letters in the row (compare viscosities).

⁎ Two-way ANOVA adjusted by bootstrapping.

## Discussion

This study aimed to investigate the impact of varying resin cement viscosities (high and low) and thicknesses (thin and thick) on their shear bond strength (s-SBS) to lithium disilicate. A significant difference in s-SBS was observed as a function of viscosity for the thick layer group (150 µm), supporting the first conceptual hypothesis. Conversely, cement thickness did not influence s-SBS as a main factor, which supports the second hypothesis.

The representative thickness ranges, 55.63 to 57.05 µm for the thin layer and 143.18 to 153.90 µm for the thick layer (Table 3), were insufficient to affect static shear strength. This suggests that increasing the resin cement volume did not generate relevant residual tensile stresses within the layer.^13^^,^^5^ This finding indicates that small variations in resin cement thickness (at the micrometre scale) likely have minimal impact on bonding outcomes, which aligns with previous reports.^15^^,^^2^^,^^4^^,^^13^^,^^9^^,^^14^^,^^5^ Clinically, this is relevant because, despite advances in ceramic manufacturing, internal and marginal misfits remain possible.^20^^,^^21^

However, when considering the performance of low-viscosity resin cement at increased thickness (L-150 group), a deleterious effect on s-SBS was observed when compared to high-viscosity resin cement (H-150 group; Table 4). Resin composite properties largely depend on material composition, and inorganic filler content affects polymerization shrinkage: lower filler content generally leads to greater shrinkage.^6^^,^^22^ The inverse relationship between filler volume and volumetric shrinkage is explained by the higher proportion of monomers available for polymerization.^23^^,^^24^ Low-viscosity resin cement has a lower filler content compared to the high-viscosity cement. In this context, shrinkage may cause bond dislodgement, increased porosity, or internal loss of cohesion.^25^ Therefore, the higher shrinkage of the low-viscosity cement may lead to premature bond failure between the resin composite and substrate, explaining the observed behaviour at greater thicknesses.^25^^,^^22^

Another important aspect is the failure pattern observed in the tests. Only the H-50 group exhibited cohesive failures, while all other groups showed adhesive failures. Additionally, the H-50 group showed a high standard deviation, which may be attributed to the seating of the restoration on the substrate. Higher viscosity materials resist flow, potentially trapping bubbles within the cement, as evidenced by microscopy images (Figure 3). The presence of these bubbles can lead to the initiation of cohesive failures when shear stresses are applied, which can influence the variability of the bond strength under these conditions. No specific analyses were performed to directly assess surface wetting or hybrid layer quality; therefore, any potential influence of cement viscosity on these aspects is considered exploratory.

A preliminary study evaluated the effect of resin cement viscosities on static and fatigue shear bond strength between lithium disilicate and dentin substrates.^7^ The thickness tested was the same as the thin layer in the present study, and no differences were found between viscosities under static or intermittent loading. Intermittent loading is important as it can induce failure through repetitive stress application. Future studies could include simulated thickness variations to further explore these effects.

Despite promising results, some limitations should be noted. A major limitation is the absence of aging procedures. Future research should include thermocycling and water storage to better challenge the bonded assemblies.^26^^,^^27^ Another limitation concerns the fatigue shear testing equipment, which is not commercially available, thereby limiting the reproducibility of the current tests. Although widely used for comparative screening, the shear bond strength test produces complex, non-uniform stress distributions at the interface, which should be considered a limitation when interpreting absolute bond strength values.

Overall, this study demonstrates that resin cement viscosity significantly influences the s-SBS of a lithium disilicate ceramic when adhering to thicker cement layers (≈150 µm), while cement with ≈50 µm has minimal influence. These findings highlight the critical role of resin cement material properties – particularly viscosity and polymerization shrinkage – in bond durability. Although limited to *in vitro* conditions, these findings enhance the understanding of how resin cement properties affect bonding performance and may guide material selection, especially in clinical scenarios where thicker cement layers may be unavoidable. From a clinical perspective, when increased cement thickness is anticipated – such as in cases of internal misfit, limited seating pressure, or complex restoration geometry – the use of higher-viscosity resin cements may be preferred to reduce the risk of reduced interfacial bond strength, based on the in vitro findings of the present study. Further studies incorporating aging protocols and fatigue testing are necessary to confirm these findings under clinically relevant conditions.

## Conclusions

When adhering lithium disilicate to thin resin cement layers (≈50 µm), the static shear bond strength is not affected by cement viscosity, indicating that small variations in viscosity have minimal influence when the cement layer is thin. In contrast, when considering thicker resin cement layers (≈150 µm), viscosity plays a crucial role, with high-viscosity providing superior shear bond strength compared to low-viscosity.

## Author Contributions

*Conceptualization*: Renan Vaz Machry, Gabriel Kalil Rocha Pereira, Kiara Serafini Dapieve, Cornelis Johannes Kleverlaan.

*Methodology*: Renan Vaz Machry, Kiara Serafini Dapieve, Ana Carolina Cadore-Rodrigues.

*Validation*: Gabriel Kalil Rocha Pereira, Luiz Felipe Valandro, Cornelis Johannes Kleverlaan.

*Formal Analysis*: Renan Vaz Machry, Kiara Serafini Dapieve.

*Investigation*: Renan Vaz Machry, Kiara Serafini Dapieve, Ana Carolina Cadore-Rodrigues.

*Resources*: Luiz Felipe Valandro, Cornelis Johannes Kleverlaan.

*Data Curation*: Renan Vaz Machry.

*Writing – Original Draft Preparation*: Renan Vaz Machry, Kiara Serafini Dapieve.

*Writing – Review & Editing*: João Paulo Mendes Tribst, Amanda Maria de Oliveira Dal Piva, Cornelis Johannes Kleverlaan.

*Visualization*: João Paulo Mendes Tribst.

*Supervision*: Luiz Felipe Valandro, Cornelis Johannes Kleverlaan.

*Project Administration*: Luiz Felipe Valandro, Cornelis Johannes Kleverlaan, João Paulo Mendes Tribst, Gabriel Kalil Rocha Pereira.

*Funding Acquisition*: Luiz Felipe Valandro, Cornelis Johannes Kleverlaan, Gabriel Kalil Rocha Pereira.

## Conflict of interest

The authors declare that they have no known competing financial interests or personal relationships that could have appeared to influence the work reported in this paper.
